# A simple approach to prevent skin damage from heat steam in robotic breast surgery

**DOI:** 10.3389/fonc.2025.1580504

**Published:** 2025-05-16

**Authors:** Kuo Chen, Pengwei Lu

**Affiliations:** Department of Breast Surgery, The First Affiliated Hospital of Zhengzhou University, Zhengzhou, China

**Keywords:** robotic surgery, breast reconstruction, skin, heat damage, gel implant

## Abstract

**Objective:**

To prevent heat-steam-induced skin damage during robotic nipple-sparing mastectomy with immediate breast reconstruction (RNSMIBR). Methods: Clinical data from 156 breast cancer patients undergoing RNSMIBR were analyzed retrospectively. Patients were divided into three groups: no skin cooling (group A, 29 cases), gauze cooled by ice water (group B, 99 cases), and gauze with ice water combined with suction tubing (group C, 28 cases). Key parameters such as age, BMI, and cancer pathology showed no significant differences across groups (P>0.05). Intraoperative skin temperature, mastectomy duration, and complications from heat-steam-induced skin damage were recorded.

**Results:**

Groups B and C had significantly lower skin temperatures compared to group A (P<0.01). Heat-steam skin damage occurred in seven cases: five in group A and two in group B (P<0.05). One group A patient experienced severe complications, requiring implant removal.

**Conclusion:**

Cooling breast skin with ice-water-soaked gauze combined with suction tubing during RNSMIBR significantly reduces the risk of heat-steam-induced skin damage.

## Introduction

Traditional breast reconstruction surgery techniques are relatively mature now, but due to the need to make a long incision on the surface of the breast, it is easy to cause greater trauma, increase the risk of infection, bleeding and flap necrosis, resulting in prolonged hospitalization, and in some serious cases, the need for a second surgery to remove the implants, increasing the patient’s pain and medical costs, and even reconstruction surgery failure; in addition, the incision on the surface of the breast inevitably form a significant scar. With the updating of medical equipment and advances in surgical technology, an increasing number of patients and doctors are opting for minimally invasive lumpectomy and robotic surgery methods that are less invasive, have fewer complications, and yield better cosmetic results. The da Vinci robotic surgical system (Intuitive Surgical Corp., Sunnyvale, CA, USA) has a high-definition, three-dimensional, and microscopically magnified field of view, and a 540° rotating robotic arm that allows for precise manipulation in confined spaces (1). Many clinical applications have shown that it has been applied in breast surgery, which has led to the development of robotic nipple-sparing mastectomy and immediate breast reconstruction with gel implant(RNSMIBR), which not only has the same oncologic safety but also better cosmetic and psychosocial outcomes compared with traditional breast reconstruction (2–4).

The use of inflatable technology in lumpectomy breast reconstruction surgery has become widespread (5, 6). In contrast to the natural space of the abdominal cavity, the breast is solid tissue. Therefore, the limited operating space in RNSMIBR surgery must be maintained by filling it with constant air pressure of CO_2_. The use of bipolar and electric scissors for the separation of the luminal space and removal of the mammary glands in RNSMIBR surgery generates a substantial amount of heat steam, which fills the entire operating space very quickly. This results in the accumulation of heat over a short period in the confined space inside the breast. This causes a large amount of heat to build up in a small space inside the breast within a short period of time. As this heat steam cannot be discharged in time, the temperature in the breast space increases rapidly, resulting in thermal heat steam damage to the breast skin and subcutaneous tissues, causing skin complications. However, to maintain a stable air pressure and avoid the outflow of CO_2_ gas, it is impossible to open the airway to allow the outflow of hot steam during the operation. To solve the above problems, we propose to cover the breast surface with ice-water-cooled gauze during RNSMIBR mastectomy and use our own adjustable suction tubes to extract the hot steam, which reduces the temperature by physical cooling, thus reducing the occurrence of hot steam damage to the skin. This retrospective comparative study was conducted to investigate the effectiveness of this method.

## Materials and methods

### Patients

Inclusion criteria for patients: 1. Female patients with confirmed unilateral breast cancer. 2. Patients who are not eligible for breast-conserving surgery according to the National Comprehensive Cancer Network (NCCN). 3. Imaging examination confirms that the breast cancer shows no evidence of multiple lymph node metastasis and no evidence of nipple, skin, or chest wall invasion. 4. The patient is willing to undergo the RNSMIBR surgical treatment. Exclusion criteria for patients: 1. Anaphylactic reaction to anesthetic drugs. 2. Mental illness and a combination of severe cardiac, pulmonary, hepatic, and renal insufficiency, as well as other contraindications to surgery. 3. A history of substance abuse. A total of 156 patients met the selection criteria for inclusion in this study between September 2022 and May 2024. The breasts were not treated in 29 cases (group A), covered with gauze cooled by ice water to reduce skin temperature in 99 cases (group B), and combined with suction tubing in 28 cases (group C). The age of the patients in the three groups, side of the operation, body mass index, pathological type of breast cancer, and percentage of patients who received adjuvant chemotherapy and neoadjuvant chemotherapy were compared. A ratio comparison of adjuvant chemotherapy revealed no statistically significant differences (P > 0.05) (**Table 1**). The surgical procedures were conducted with the da Vinci XI robotic surgical system (Intuitive Surgical Corp., Sunnyvale, CA, USA), and all patients received Mentor anatomical Gel implants (Mentor Worldwide LLC, USA). Data were recorded and managed using Microsoft Excel software (Microsoft Corp., Redmond, WA, USA). This study involved human participants and was conducted in full compliance with ethical standards. Ethical approval was obtained from the Institutional Review Board of Chinese Clinical Trial Registry (ChiCTR) (ChiCTR2500096970). Prior to inclusion in the study, all patients were fully informed of the nature, purpose, potential risks, and benefits of the procedure. Written informed consent was obtained from each participant. All surgical procedures were performed by the same team of experienced breast surgeons, who had no conflict of interest in surgical decision-making and maintained full autonomy in their clinical judgment. Each surgeon had completed comprehensive robotic surgical training, including simulator-based and animal model practice, at the official training center of Intuitive Surgical.

**Table 1:** Comparison of baseline data between the 3 groups.

Baseline data	Group A (N=29)	Group B (N=99)	Group C (N=28)	Statistical value	P value
Age (*x±s*, year)	39.07±4.60	42.00±8.96	41.04±8.65	F= 1.25	0.289
*BMI (x±s, kg/m2)*	22.31±2.19	23.44±3.15	23.26±2.09	F=3.84	0.023
Location (*Right/Left, N*)	17/12	51/48	17/11	*χ2=*0.99	0.609
Pathological type (*DCIS/ IDC, N*)	9/20	19/80	8/20	*χ2=*2.35	0.308
AC (N,%)	14 (48.3)	63 (63.6)	14 (50.0)	*χ2=*1.31	0.519
NAC (N,%)	6 (20.7)	17 (17.2)	6 (21.4)	*χ2=*0.30	0.862
Breast reconstruction (*Gel implant/ Gel implant+CDM*)	21/8	94/5	16/12	*χ2=*26.72	0.000

DCIS, Ductal carcinoma in situ; IDC, Invasive ductal carcinoma; AC, Adjuvant chemotherapy; NAC, Neoadjuvant chemotherapy; CBM, Contralateral breast mammaplasty.

### Surgical techniques and breast skin cooling methods

**Figure 1:**
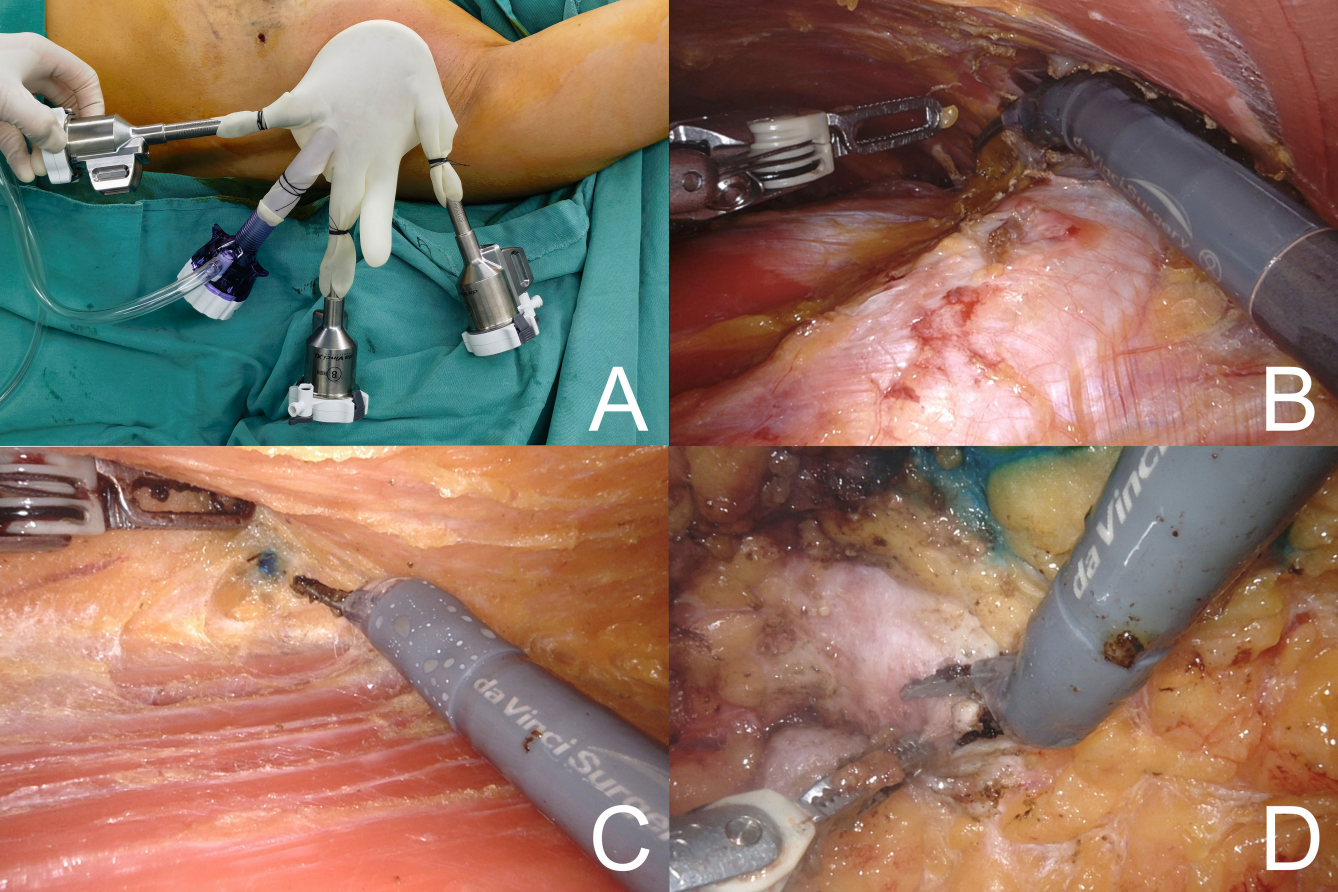
**(A)** Location of surgical incision. **(B)** Create the space for the posterior pectoralis muscle implant. **(C)** Peeling the gland and finding the edges of the gland marked with methylene blue. **(D)** Placement of the robotic arm to perform the posterior cut edge of the nipple.

The surgical procedure utilized the da Vinci Xi robotic system (Intuitive Surgical, USA). Under general anesthesia, the patient was placed in the supine position with a shoulder pad positioned beneath the affected scapula. The affected upper limb was abducted, elevated, and flexed to rest against its forehead. The incision was strategically located midway between the upper outer border of the breast and the axilla, measuring approximately 5–6 cm in length. To create the initial cavity for robotic surgery, the skin and subcutaneous tissues were incised, forming a space with approximate dimensions of 5 × 5 × 5 cm. A single-hole incision protection sleeve was applied to the site, and the robotic arm was carefully positioned, adjusted, and connected to the surgical instruments and the insufflation device (**Figure 1A**). Throughout the procedure, the breast cavity was maintained at 1.30–1.60 kPa (10–12 mmHg) using the AirSeal^®^ system. Methylene blue was injected at the periphery of the gland at the 3, 6, 9, and 12 o’clock positions to facilitate precise anatomical localization. The pectoralis major and minor intermuscular spaces were initially released with dissection along the inferior crease of the pectoralis major muscle to create the posterior pectoral implant space. This step was relatively straightforward because of the clear anatomical structures and their posterior position, minimizing the generation of hot steam and reducing the risk of skin damage to the breast (**Figure 1B**). Access to the posterior breast space was achieved via the surface of the pectoralis major muscle, which extended to the periphery of the gland. Robotic instruments were utilized to navigate the fascia plane of Scarpa, preserving a subcutaneous fat layer approximately 1 cm thick. The gland was progressively excised at marked methylene blue sites, and the flap was dissected to the edge of the gland (**Figure 1C**). The posterior edge of the nipple was excised and subjected to a pathological analysis (**Figure 1D**). Sentinel lymph node biopsy or axillary dissection was performed based on preoperative examination findings and diagnostic considerations.

**Figure 2:**
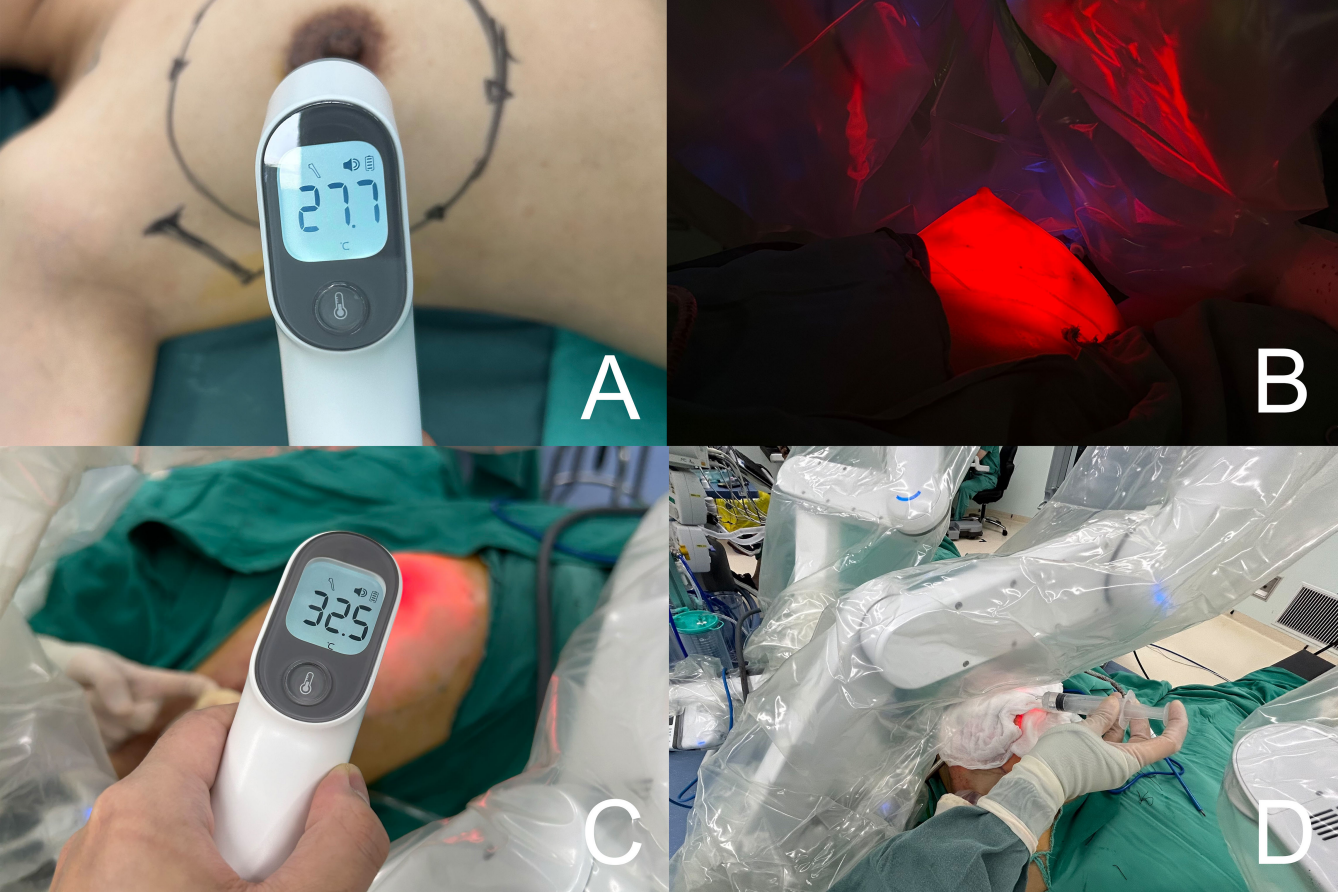
**(A)** Preoperative of breast surface temperature. **(B)** Visualizing the approximate extent of the gland excision and the thickness of the fat by using the transmittance of the robotic light source. **(C)** Surgical gauze soaked in ice water was applied to the breast surface. **(D)** Intraoperative measurement of breast surface temperature.

**Figure 3:**
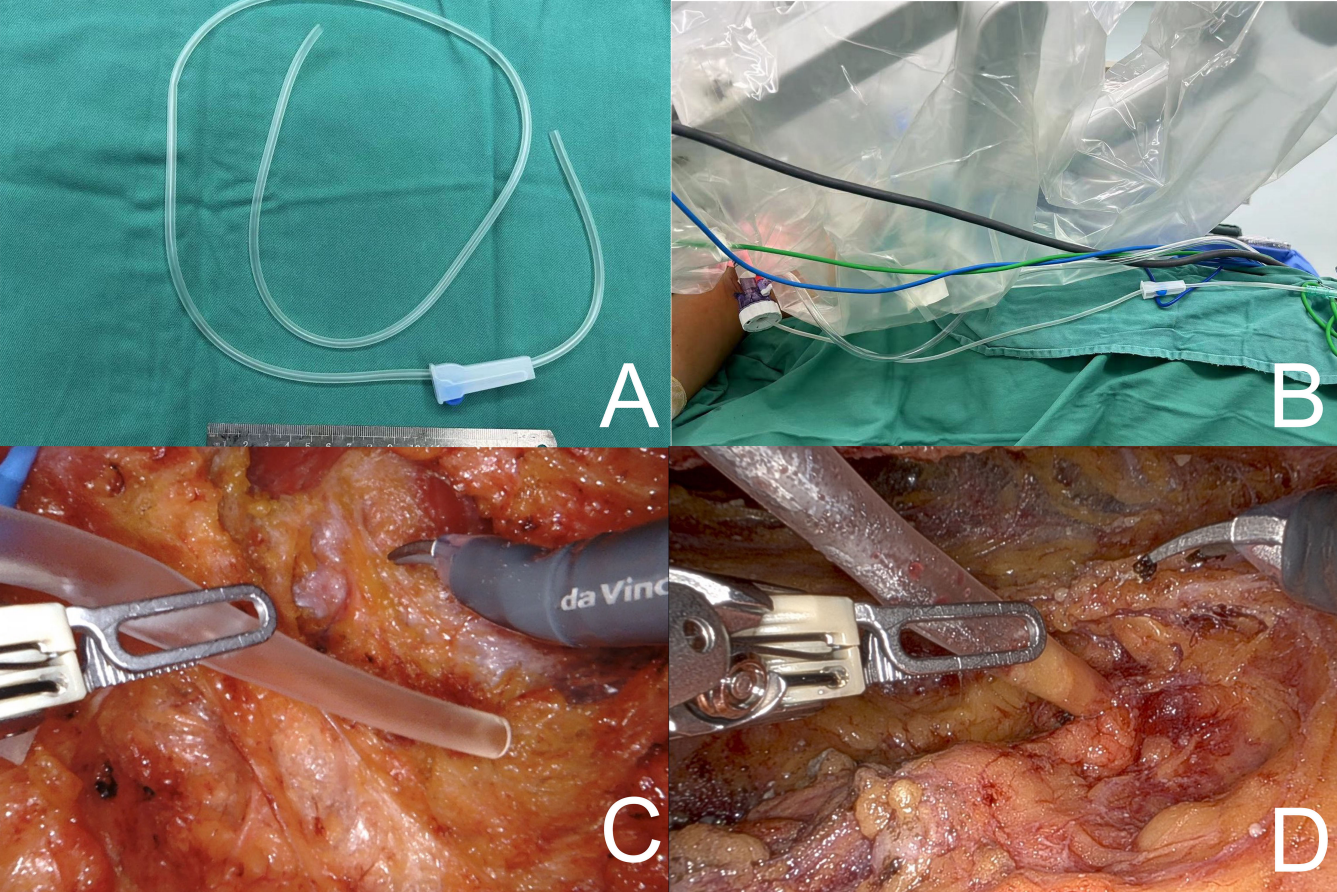
**(A, B)** A suction device was constructed using a transfusion hose. **(C)** The suction device could be moved with a grasping forceps. **(D)** The suction device could be “fixed” to the adipose tissue layer during the excision operation with the help of suction.

Bipolar grasping forceps and monopolar electroshears were employed to perform breast adenomectomy, with the robotic surgical system selected for bipolar electroshearing electrocoagulation at 45 W and monopolar unipolar electroshearing electrocoagulation at 30 W. During adenomectomy, Group A did not undergo any interventional temperature manipulation. In Group B, surgical gauze soaked in ice water was applied to the breast surface (**Figures 2A-D**). In Group C, after the breast surface was covered with gauze, a suction device was constructed using a transfusion hose. This was connected to an inflatable device and an external suction device was attached (**Figures 3A, B**). The suction device could be moved with grasping forceps to remove smoke and heat steam during operation (**Figure 3C**). However, continuous suction results in a decrease in CO2 pressure, which could affect normal operation. Therefore, the suction device could be “fixed” to the adipose tissue layer during the excision operation with the help of suction and subsequently picked up with the grasping forceps during the procedure (**Figure 3D**). Implementation of the AirSeal^®^ system during surgical procedures facilitates the maintenance of a stable breast space, ensuring consistent pressure within the operative field.

A thermometer was employed to ascertain the temperature change of the breast surface, and the average value was derived from five measurements. Following gland removal, the approximate extent of excision was observed through the translucency of the robotic light source (**Figure 2B**). Subsequently, the post-glandular excision space was examined and the gland specimen was removed through an incision in the axilla. The robotic surgical instruments were then removed and the time taken for unilateral RNSM was recorded. The surgical site was thoroughly irrigated, bilateral breast dimensions were evaluated, and the implant was positioned behind the pectoralis major muscle. A Mentor^®^ anatomical silicone gel implant (Mentor Corporation, USA) was used in this cohort. Two drainage tubes were retained *in situ* and the incision was closed in a layered manner.

### Indicators are employed for the purpose of evaluating the extent of hot steam damage to breast skin

It is inaccurate to define counting time as the interval between the setup of the robotic surgical platform, activation of the surgical instruments, completion of the robotic mastectomy, and removal of the mastectomy specimen. This is because subject to the learning curve, this time includes processes such as instrument arm adjustment and cleaning of the lens, which consume a significant amount of time during the initial stages of the learning curve, during which no gland removal operation is performed; thus, no heat steam is generated. Accordingly, the RNSM time affecting the breast skin was defined as the approximate time at which the gland was removed using the robotic instrument arm.

Currently, direct measurement of the temperature within the breast operating space is not feasible, as commercially available thermometers are not compatible with autoclave sterilization protocols required for intraoperative use. Therefore, a standardized non-invasive surface temperature measurement method was employed. Five readings were taken at five anatomically fixed locations on the breast surface using a handheld infrared thermometer. To ensure consistency, the distance between the thermometer and each measurement site was maintained at 5 cm using a custom-designed spacer, and measurements were taken perpendicular to the skin surface. Each site was measured three times, and the average of these readings was used to calculate the overall mean surface temperature of the breast. To enhance measurement reliability, the contact thermocouple probes were encased in sterile protective covers to ensure intraoperative sterility. Parallel measurements were conducted using these contact probes, and the results showed high consistency with those obtained from the infrared thermometer, with a deviation within ±0.3°C. This cross-validation supports the accuracy and validity of our surface temperature assessment method.

The analysis was conducted using IBM SPSS Statistics for Windows version 26.0. The data were found to conform to a normal distribution according to the Kolmogorov-Smirnov test and were expressed as mean ± standard deviation. An independent samples t-test was used to compare two groups. Similarly, the chi-square test was used to compare the count data in the table. The test level was set at α = 0.05.

## Results

**Figure 4:**
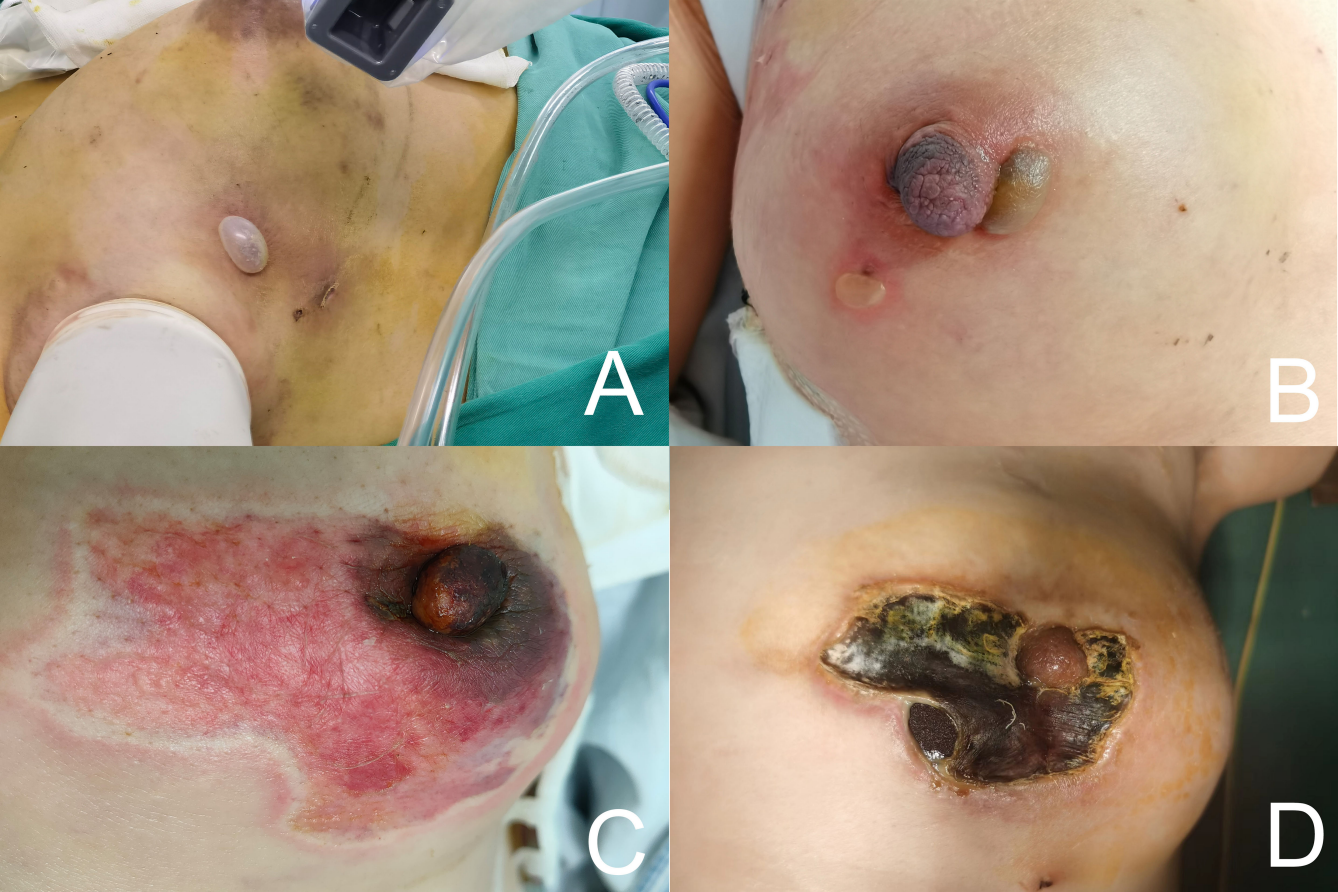
**(A, B)** Blisters and erythematous flaps formed after intraoperative heat steam skin damage. **(C, D)** Exposure of the implant after the scab fell off at 1 month after heat steam skin damage.

The mean time for the unilateral gland removal operation in groups A (76.38 ± 12.88) minutes, B (77.18 ± 9.23) minutes, C (68.39 ± 5.78) minutes. The difference between the three groups was statistically significant MD (95% CI) = 74.08 (± 1.82) min; P< 0.001). The intraoperative breast surface temperatures in groups B and C were (25.61 ± 0.91)°C and 24.94 ± 1.17)°C, which were lower than those in group A (33.38 ± 1.14)°C, and the differences were also statistically significant [MD (95% CI)=26.94 (± 0.51)°C; P<0.001]. Seven cases of heat steam skin damage occurred during the operation, including two cases (2.0%) in group B and five cases (17.2%) in group A, with a significant difference in incidence between the three groups (P<0.05). Among them, 1 patient in group A had blister rupture and infection, which eventually led to the removal of the implant; the rest of the patients were treated with postoperative interventions for skin recovery (**Table 2**) (**Figures 4A-D**).

**Table 2:** Postoperative skin complications related to the heat steam induced damage between the 3 groups.

Indicators of results		Group A (N=29)	Group B (N=99)	Group C (N=28)	Median deviation (95% Confidence Interval)	P value
Time for the unilateral gland removal operation ( $\bar{x}\;\pm \;s$ , min)		76.38±12.88	77.18±9.23	68.39±5.78	MD=74.08 (±1.82)	0.000**
Intraoperative breast skin surface temperature ( $\bar{x}\;\pm \;s$ , °C)		33.38±1.14	25.61±0.91	24.94±1.17	MD=26.94 (±0.51)	0.000**
Skin complications{n(%)}	Erythematous flaps and vesication	5(12.7)	2(2.0)	0(0)	A vs B: OR = 10.10 A vs C: OR ≈ 12.79	0.0010
	Loss of implant	1(3.45)	0(0)	0(0)	A vs B: OR ≈ 10.47 A vs C: OR ≈ 3.00	0.1104

** p<0.01.

Importantly, all patients underwent a routine clinical follow-up one month after surgery. No delayed complications, such as skin necrosis, delayed wound healing, or implant-related issues, were observed in any of the three groups. These findings suggest that while intraoperative heat-steam damage may pose acute risks, its long-term impact can be minimized with prompt recognition and effective postoperative management.

## Discussion

The increasing popularity of laparoscopic and robotic surgeries has led to the widespread adoption of insufflation as a means of creating an operating space. The insufflation method is employed to maintain the operating space of the breast by filling it with CO2 without destroying the relevant anatomical structures. Gunusen et al. (7) conducted a comparative study on the effects of CO2 pneumoperitoneum at different temperatures and humidities on hemodynamic and respiratory parameters during gynecological laparoscopy and postoperative pain. The results demonstrated that heated and humidified CO2 had no effect on the hemodynamic and respiratory parameters of patients. However, the patient’s core body temperature was elevated and an inflammatory response was observed. A meta-analysis by Sharma et al. (8) also showed that patients undergoing colorectal surgery who received heated humidified CO2 did not experience a clinically meaningful difference in core body temperature change compared with their counterparts who received dry-cold CO2 insufflation or no insufflation.

Toesca et al. (9) reported that a patient undergoing RNSMIBR developed a minor blistering effect from internal electrocautery of the breast skin flap. Lai et al. (10) demonstrated that the incidence of blister formation in RNSM was 2.6% (N=76), which was attributed to the observation that breast mastectomy is performed at a slower rate during the initial phase of the learning curve, resulting in a reduction in heat generation over time. Subsequently, if mastectomy is conducted at a faster rate following the learning curve, a considerable quantity of heat steam accumulates in a relatively brief period. However, compared to the extensive anatomical space of the abdominal cavity, the breast space is relatively limited. The heat steam generated by surgical instruments cutting through tissues accumulates in significant quantities within a relatively short period, resulting in thermal damage to the breast skin. This is particularly evident when patients are in a supine position, as the upward evaporation of heat steam increases the risk of injury to the nipple areola, which is situated in the highest position and is the most vulnerable area of subcutaneous fat.

The RNSMIBR procedure commences with separation of the posterior pectoralis major space and the surface plane of the pectoralis major muscle, where thermal heat steam damage to the skin is minimal. Most thermal injuries occur at the subcutaneous level of separation, necessitating particular attention during the procedure. Therefore, RNSM time was redefined and recorded in this study. The use of a reverse sequence approach to the surgical procedure theoretically mitigates the risk of thermal skin injuries. Furthermore, intraoperative separation from the surface of the gland in the plane of the scarpa fascia and preservation of the large subcutaneous fat layer are also conducive to the protection of the breast skin. In a study conducted by Cooper et al. (11), it was demonstrated that esophageal cooling during radiofrequency ablation not only reduced the risk of intraoperative esophageal injury but also significantly reduced the time required for the procedure and the amount of postoperative pain experienced by patients. Intraoperative application of ice saline, a straightforward technique for the management of hepatocellular carcinoma, has also been demonstrated to effectively mitigate local tissue damage (12, 13).

In this retrospective study, we proposed the use of gauze covered with ice water to reduce breast skin temperature during RNSMIBR and constructed a simple suction device to investigate its effectiveness. The AirSeal system was used to maintain intraoperative air pressure stabilization. Concurrently, the suction apparatus was affixed to the adipose tissue via a robotic arm. Concurrently, the device can be utilized by maneuvering it through a robotic arm to serve as an “on/off” switch, when required. The findings of this study demonstrated that the intraoperative breast surface temperatures and the incidence of thermal injury were markedly reduced in groups B and C compared to the untreated group A. This indicates that the proposed straightforward method is effective. These findings indicate that this straightforward approach is efficacious, and that skin heat steam injury and associated severe complications can be averted through prompt intervention. Nevertheless, this study had some limitations. The measurement of breast surface temperature using a thermometer is not accurate, and the temperature within the confined space of the breast itself cannot be accurately gauged. Therefore, further studies are warranted.

In light of this, a well-designed prospective, randomized controlled trial could offer more robust evidence. Such a study might involve real-time temperature monitoring with sterilizable sensors placed closer to the surgical cavity, standardized application of cooling protocols, and a longer follow-up period to assess late-onset complications or aesthetic outcomes. Stratification based on patient-specific factors such as BMI, skin thickness, or breast volume would also enhance the generalizability of findings.

## Conclusions

In conclusion, intraoperative cooling of the breast is essential to prevent heat steam damage to the breast skin in RNSMIBR. This paper presents a preliminary investigation of the efficacy of various cooling techniques. Further optimization of these techniques is necessary to develop strategies for the prevention and treatment of postoperative skin complications in patients undergoing robotic breast surgery.

## Data Availability

The data analyzed in this study was obtained from the First Affiliated Hospital of Zhengzhou University, the following licenses/restrictions apply: The data that support the findings of this study were used under license from the First Affiliated Hospital of Zhengzhou University for the current study, and thus are not publicly available. However, data were available from the authors upon reasonable request and with permission from the First Affiliated Hospital of Zhengzhou University. Requests to access these datasets should be directed to Kuo Chen, chenkchenk@foxmail.com.

## References

[1] Chen K Zhang J Beeraka NM Song D Sinelnikov MY Lu P. Robot-assisted nipple-sparing mastectomy and immediate breast reconstruction with gel implant and latissimus dorsi muscle flap: Our initial experience. Int J Med Robot. (2023) 19:e2528. doi: 10.1002/rcs.v19.5 37194617

[2] Toesca A Peradze N Galimberti V Manconi A Intra M Gentilini O. Robotic nipple-sparing mastectomy and immediate breast reconstruction with implant: first report of surgical technique. Ann Surg. (2017) 266(2):e28–30. doi: 10.1097/SLA.0000000000001397 28692558

[3] Chen K Zhang J Beeraka NM Lu P. Robotic nipple sparing mastectomy and immediate breast reconstruction: significant attempts with the latissimus dorsi muscle without island flap. Minerva Surg. (2024) 79:411–8. doi: 10.23736/S2724-5691.24.10244-4 38757888

[4] Toesca A Park HS Ryu JM Kim YJ Lee J Sangalli C. Robot-assisted mastectomy: next major advance in breast cancer surgery. Br J Surg. (2023) 110(4):502–3. doi: 10.1093/bjs/znad006 36708043

[5] Hwang YJ Chung JH Lee HC Park SH Yoon ES. Single-port transaxillary robot-assisted latissimus dorsi muscle flap reconstruction for Poland syndrome: concomitant application of robotic system to contralateral augmentation mammoplasty. Arch Plast Surg. (2022) 49:373–7. doi: 10.1055/s-0042-1748647 PMC9142219 35832149

[6] Ryu JM Kim JY Choi HJ Ko B Kim J Cho J. Robot-assisted nipple-sparing mastectomy with immediate breast reconstruction: an initial experience of the korea robot-endoscopy minimal access breast surgery study group (KoREa-BSG). Ann Surg. (2022) 275(5):985–91. doi: 10.1097/SLA.0000000000004492 32941285

[7] Gunusen I Akdemir A Sargın A Karaman S. The effects of CO(2) pneumoperitoneum at different temperature and humidity on hemodynamic and respiratory parameters and postoperative pain in gynecological laparoscopic surgery: A prospective randomized controlled study. Asian J Surg. (2022) 45:154–61. doi: 10.1016/j.asjsur.2021.04.005 33888367

[8] Sharma S McKechnie T Khamar J Wu K Hong D Eskicioglu C. The role of warmed-humidified carbon dioxide insufflation in colorectal surgery: A systematic review and meta-analysis. Colorectal Dis. (2024) 26:7–21. doi: 10.1111/codi.16798 37985859

[9] Toesca A Peradze N Manconi A Galimberti V Intra M Colleoni M. Robotic nipple-sparing mastectomy for the treatment of breast cancer: Feasibility and safety study. Breast. (2017) 31:51–6. doi: 10.1016/j.breast.2016.10.009 PMC5278881 27810700

[10] Lai HW Chen DR Liu LC Chen ST Kuo YL Lin SL. Robotic versus conventional or endoscopic-assisted nipple-sparing mastectomy and immediate prosthesis breast reconstruction in the management of breast cancer: A prospectively designed multicenter trial comparing clinical outcomes, medical cost, and patient-reported outcomes (RCENSM-P). Ann Surg. (2024) 279(1):138–46. doi: 10.1097/SLA.0000000000005924 PMC10727200 37226826

[11] Cooper J Joseph C Zagrodzky J Woods C Metzl M Turer RW. Active esophageal cooling during radiofrequency ablation of the left atrium: data review and update. Expert Rev Med Devices. (2022) 19(12):949–57. doi: 10.1080/17434440.2022.2150930 PMC9839561 36413154

[12] Ogawa T Kawamoto H Kobayashi Y Nakamura S Miyatake H Harada R. Prevention of biliary complication in radiofrequency ablation for hepatocellular carcinoma-Cooling effect by endoscopic nasobiliary drainage tube. Eur J Radiol. (2010) 73(2):385–90. doi: 10.1016/j.ejrad.2008.10.021 19056192

[13] Nakata Y Haji S Ishikawa H Yasuda T Nakai T Takeyama Y. Two cases of hepatocellular carcinoma located adjacent to the Glisson’s capsule treated by laparoscopic radiofrequency ablation with intraductal chilled saline perfusion through an endoscopic nasobiliary drainage tube. Surg Laparosc Endosc Percutan Tech. (2010) 20(6):e189–92. doi: 10.1097/SLE.0b013e3181f91ba2 21150399

